# Pharokka: a fast scalable bacteriophage annotation tool

**DOI:** 10.1093/bioinformatics/btac776

**Published:** 2022-12-01

**Authors:** George Bouras, Roshan Nepal, Ghais Houtak, Alkis James Psaltis, Peter-John Wormald, Sarah Vreugde

**Affiliations:** Adelaide Medical School, Faculty of Health and Medical Sciences, The University of Adelaide, Adelaide, SA 5070, Australia; Department of Surgery—Otolaryngology Head and Neck Surgery, University of Adelaide and the Basil Hetzel Institute for Translational Health Research, Central Adelaide Local Health Network, Adelaide, SA 5070, Australia; Adelaide Medical School, Faculty of Health and Medical Sciences, The University of Adelaide, Adelaide, SA 5070, Australia; Department of Surgery—Otolaryngology Head and Neck Surgery, University of Adelaide and the Basil Hetzel Institute for Translational Health Research, Central Adelaide Local Health Network, Adelaide, SA 5070, Australia; Adelaide Medical School, Faculty of Health and Medical Sciences, The University of Adelaide, Adelaide, SA 5070, Australia; Department of Surgery—Otolaryngology Head and Neck Surgery, University of Adelaide and the Basil Hetzel Institute for Translational Health Research, Central Adelaide Local Health Network, Adelaide, SA 5070, Australia; Adelaide Medical School, Faculty of Health and Medical Sciences, The University of Adelaide, Adelaide, SA 5070, Australia; Department of Surgery—Otolaryngology Head and Neck Surgery, University of Adelaide and the Basil Hetzel Institute for Translational Health Research, Central Adelaide Local Health Network, Adelaide, SA 5070, Australia; Adelaide Medical School, Faculty of Health and Medical Sciences, The University of Adelaide, Adelaide, SA 5070, Australia; Department of Surgery—Otolaryngology Head and Neck Surgery, University of Adelaide and the Basil Hetzel Institute for Translational Health Research, Central Adelaide Local Health Network, Adelaide, SA 5070, Australia; Adelaide Medical School, Faculty of Health and Medical Sciences, The University of Adelaide, Adelaide, SA 5070, Australia; Department of Surgery—Otolaryngology Head and Neck Surgery, University of Adelaide and the Basil Hetzel Institute for Translational Health Research, Central Adelaide Local Health Network, Adelaide, SA 5070, Australia

## Abstract

**Summary:**

In recent years, there has been an increasing interest in bacteriophages, which has led to growing numbers of bacteriophage genomic sequences becoming available. Consequently, there is a need for a rapid and consistent genomic annotation tool dedicated for bacteriophages. Existing tools either are not designed specifically for bacteriophages or are web- and email-based and require significant manual curation, which makes their integration into bioinformatic pipelines challenging. Pharokka was created to provide a tool that annotates bacteriophage genomes easily, rapidly and consistently with standards compliant outputs. Moreover, Pharokka requires only two lines of code to install and use and takes under 5 min to run for an average 50-kb bacteriophage genome.

**Availability and implementation:**

Pharokka is implemented in Python and is available as a bioconda package using ‘conda install -c bioconda pharokka’. The source code is available on GitHub (https://github.com/gbouras13/pharokka). Pharokka has been tested on Linux-64 and MacOSX machines and on Windows using a Linux Virtual Machine.

## 1 Introduction

As the number of bacteriophage (phage) sequences increases, there is a need for bioinformatic tools that enable fast, consistent and scalable genomic annotation (Cook *et al.*, 2021). Existing tools such as RAST (Aziz *et al.*, 2008; Davis *et al.*, 2020), PHASTER (Arndt *et al.*, 2016) and CPT Galaxy (Ramsey *et al.*, 2020) are web-server based which may be laborious in particular when multiple phage genomes require annotation (Shen and Millard, 2021). On the other hand, currently available customizable bioinformatics pipelines such as multiPhATE2 require significant understanding of bioinformatics to implement and have lengthy run times (Ecale Zhou *et al.*, 2021). Furthermore, command-line programs designed for viral discovery in metagenomic datasets such as Cenote-Taker 2 (Tisza *et al.*, 2021), Hecatomb (Roach *et al.*, 2022) and MetaPhage (Pandolfo *et al.*, 2022) require significant computational resources and storage for database installation.

As a result, one-line prokaryotic genome annotation tools such as Prokka (Seemann, 2014) are often used for phages, especially where tens or hundreds of phages need to be annotated simultaneously (Beamud *et al.*, 2022; Nordstrom *et al.*, 2022). However, such tools implement prokaryotic gene prediction tools that are based on models that are not designed for phage genomes. In addition, phage genes are often lacking in their default databases, resulting in incomplete or hypothetical functional annotation of phage genes.

Inspired by Prokka, here we created Pharokka as a one-line tool tailored to phages that provides annotations in a fast, scalable and consistent fashion. Pharokka identifies predicted coding sequences (CDS), transfer RNAs (tRNAs), transfer-messenger RNAs (tmRNAs) and clustered regularly interspaced short palindromic repeats (CRISPRs), providing functional annotation for CDS using the PHROGs database (Terzian *et al.*, 2021). With accessible bioconda installation, Pharokka is easily integrated into complex bioinformatic pipelines such as those created with Snakemake (Mölder *et al.*, 2021) or Nextflow (Di Tommaso *et al.*, 2017).

## 2 Materials and methods

**Fig. 1.**
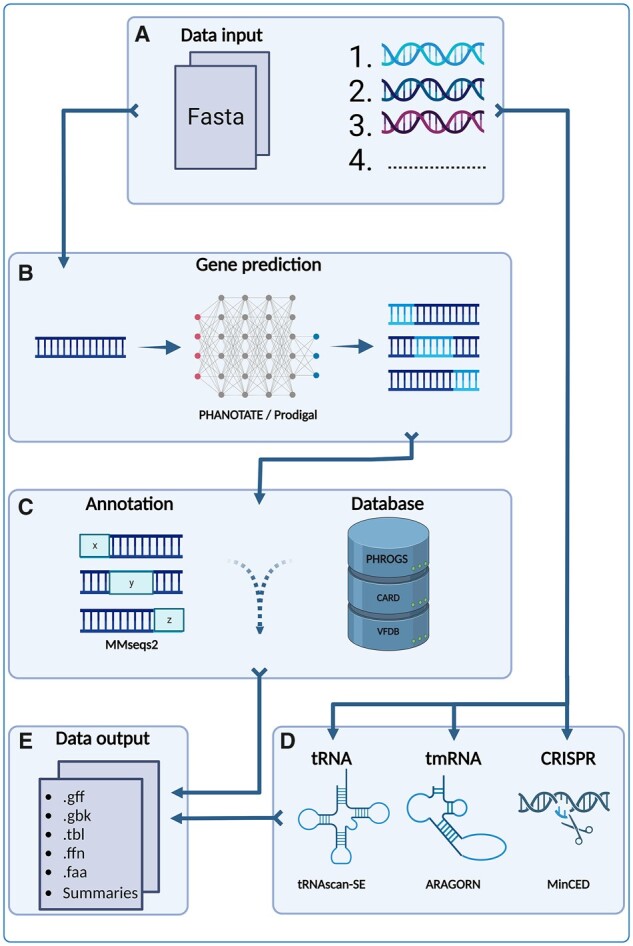
Pharokka workflow. (**A**) An input phage complete assembly or input phage contigs are loaded. (**B**) CDS are predicted with PHANOTATE (default) or Prodigal. (**C**) CDS are functionally annotated by matching them to the PHROGs database with mmseqs2. The CDS are then matched to the CARD and the VFDB. (**D**) tRNAs, tmRNAs and CRISPRs are detected using tRNAscan-SE, Aragorn and MinCED. (**E**) All annotations are amalgamated into standards compliant output formats

The Pharokka workflow is outlined in Figure 1.

### 2.1 Input

Pharokka requires assembled DNA sequences in FASTA format (Fig. 1A). For phage isolates, this usually consists of one complete contig, but Pharokka can also annotate incomplete assemblies or metavirome samples with multiple contigs in the multiFASTA format. Furthermore, metagenomically assembled phage genomes and genomic contigs, derived using programs such as Virstorter2 (Guo *et al.*, 2021), Hecatomb (Roach *et al.*, 2022) and Cenote-taker 2 (Tisza *et al.*, 2021), are also suitable to be annotated using Pharokka using meta mode.

### 2.2 Feature prediction

Pharokka uses PHANOTATE by default to predict CDS, as it is the only existing tool that is designed to predict genes in phage genomes (McNair *et al.*, 2019; Fig. 1B). PHANOTATE considers unique features of phage genomes such as small gene size, high coding density and alternative start codons (McNair *et al.*, 2019). Moreover, PHANOTATE predicts significantly more genes that are on average smaller than other gene prediction tools (McNair *et al.*, 2019). Small phage genes are more prevalent than predicted based on existing non-phage specific annotation tools and may encode for anti-CRISPR and antimicrobial resistance proteins (Fremin *et al.*, 2022). Alternatively, Pharokka users can specify Prodigal, a gene predictor designed for prokaryotic genes, be used for CDS prediction (Hyatt *et al.*, 2010; Fig. 1B). Prodigal may be useful if users wish to annotate large metavirome datasets quickly using meta mode (Table 4). In addition, Pharokka employs tRNAscan-SE 2 (Chan *et al.*, 2021) to predict tRNAs, Aragorn (Laslett and Canback, 2004) to predict tmRNAs and MinCED to predict CRISPRs (Bland *et al.*, 2007; Fig. 1D). When meta mode is specified, Pharokka runs PHANOTATE on each contig separately, leading to considerable speed improvement when multiple threads are specified.

**Table 1.** Description of Pharokka output files

Output files	Description of file contents
.gff	Gff3 feature file with all genomic annotations
.gbk	GenBank formatted feature file
.faa	FASTA file of predicted CDS (amino acid)
.ffn	FASTA file of predicted CDS (nucleotide)
minced_spacers.txt and minced.gff	MinCED CRISPR spacers and parsed CRISPR annotations
aragorn.txt and aragorn.gff	Aragorn tmRNA annotations
trnascan_out.gff	tRNAscan-SE 2 tRNA annotations
cds_functions.tsv	Functional summary counts of CDS
cds_final_merged_output.tsv	Combined output from PHROGs, VFDB and CARD
length_gc_cds_density.tsv	Summary of genome length, GC percentage, coding density
.tbl	Feature table for NCBI (BankIt) submission
top_hits_card.tsv and top_hits_vfdb.tsv	Top hits from CARD and VFDB annotations. Will contain no rows if there are no hits
terL.faa and terL.ffn	Predicted terminase large subunit sequences

**Table 2.** Benchmarking *Enterobacteria* phage Lambda (48 052 bp)

	Pharokka PHANOTATE	Pharokka Prodigal	Prokka with PHROGs
Time (min)	4.19	3.88	0.27
CDS	88	61	62
Coding density (%)	94.55	83.69	84.96
Annotated function CDS	43	37	45
Unknown function CDS	45	24	17

**Table 3.** Benchmarking *Staphylococcus* phage SAOMS1 (140 315 bp)

	Pharokka PHANOTATE	Pharokka Prodigal	Prokka with PHROGs
Time (min)	4.26	3.89	0.93
CDS	246	212	212
Coding density (%)	92.27	89.69	89.31
Annotated function CDS	92	93	92
Unknown function CDS	154	119	120

**Table 4.** Benchmarking 673 crAss-like metagenome assembled phage genomes (Yutin *et al.*, 2021)

	Pharokka PHANOTATE meta mode	Pharokka Prodigal meta mode	Prokka with PHROGs
Time (min)	106.55	11.88	252.33
Time gene prediction (min)	96.21	3.4	5.12
Time tRNA prediction (min)	1.25	1.08	0.30
Time database searches (min)	6.75	5.58	238.77
CDS	138 628	90 497	89 802
contig min coding density (%)	66.01	46.18	46.13
Contig max coding density (%)	98.86	97.85	97.07
Annotated function CDS	9341	9228	14 461
Unknown function CDS	129 287	81 269	75 341

### 2.3 Functional gene annotation

Functional assignment of predicted CDS is conducted using the PHROGs database (Terzian *et al.*, 2021; Fig. 1C). The PHROGs database contains 38 880 PHROGs (protein orthologous groups) containing 868 340 proteins from 17 473 complete genomes of viruses infecting bacteria or archaea that are grouped together using remote homology detection. Each PHROG is assigned to one of nine functional categories. Each gene within each PHROG has a specific product description if known. All predicted CDS are translated into a protein sequence and assigned to the closest matching protein and accompanying PHROG in the PHROGs database using the protein searching tool mmseqs2 (Steinegger and Söding, 2017). An e-value threshold of 10^−5^ is used by default. Users can also specify their own e-value if desired. This is particularly useful if the phage is novel with few or no similar sequences in the PHROGs database, as a lower threshold can be used to detect less similar matches. If no PHROG match is found, then the CDS is annotated as ‘No PHROG’ and ‘hypothetical protein’. All CDS that are annotated as ‘terminase large subunit’ are extracted into separate output files, as the terminase large subunit is commonly used for phylogenetic analysis (Al-Shayeb *et al.*, 2020).

### 2.4 Virulence factor and antimicrobial resistance gene detection

While virulence factors are commonly found on prophages (Fortier and Sekulovic, 2013), lytic phages rarely encode antibiotic resistance genes (Enault *et al.*, 2017; Cook *et al.*, 2021). Nonetheless, screening is required for all phages that are intended to be used for phage therapy (Shen and Millard, 2021). Pharokka adds antimicrobial resistance and virulence gene detection using the Comprehensive Antibiotic Resistance Database (CARD) (Alcock *et al.*, 2020) and the Virulence Factor Database (VFDB) (Liu *et al.*, 2019; Fig. 1C). All protein CDS are assigned to the closest matching protein in each database with mmseqs2 if the match passes strict thresholds of 80% identity over 40% coverage recommended by Enault *et al.* (2017).

## 3 Output

Pharokka’s output files are outlined in Table 1. The primary output of Pharokka is a .gff file that is suitable for use in downstream pan-genome programs such as Roary (Page *et al.*, 2015) and panaroo (Tonkin-Hill *et al.*, 2020; Fig. 1E). Other files include a .tbl file, which is a flat-file table suitable to be uploaded to the NCBI's Bankit, a cds_functions.tsv file, which includes counts of CDS, tRNAs, CRISPRs and tmRNAs and CDS within each functional category for each contig in the input FASTA file, a length_gc_cds_density.tsv file, which outputs the length, GC percentage and CDS coding density for each contig, a cds_final_merged_output.tsv, which gives the combined parsed output from mmseqs2 searches against the PHROGs, VFDB and CARD databases, and terL.faa and terL.ffn output files that contain the amino acid and nucleotide sequences of any predicted terminase large subunit genes. When run on metavirome input, Pharokka’s contig-level summary output allows users to identify specific contigs within the metavirome that possess unusual features such as virulence factors, antimicrobial resistance genes or potential stop codon reassignment as indicated by low CDS coding density (Peters *et al.*, 2022).

## 4 Results

To test the performance of Pharokka, we compared the run-time and annotations of Enterobacteria phage lambda (Genbank accession J02459), *Staphylococcus* phage SAOMS1 (Genbank accession MW460250) and 673 crAss-like metagenome-assembled phage genomes from the human gut (Yutin e*t al*., 2021) with default Pharokka v1.1.0 using PHANOTATE as a gene predictor, Pharokka v1.1.0 specifying Prodigal as gene predictor and Prokka v1.14.6 using a version of the PHROGs HMM database that has been reformatted for use with Prokka (Millard, 2021https://millardlab.org/2021/11/21/phage-annotation-with-phrogs/). For the 673 crAss-like phage genomes, Pharokka’s meta mode was employed. Benchmarking was conducted on an Intel^®^ Xeon^®^ CPU E5-4610 v2 @ 2.30 GHz specifying 16 threads for Pharokka and 16 cpus for Prokka.

Tables 2 and 3 show that for phages Lambda and SAOMS1, Pharokka is slower than Prokka but still fast, finishing within 5 min regardless of gene predictor used, with PHANOTATE predicting more CDS with higher coding density than Prodigal. Table 4 shows that for the crAss-like phage genomes, Pharokka is considerably faster than using Prokka regardless of gene predictor, due to Pharokka using mmseqs2 for database searching rather than HMMER3 (Finn *et al.*, 2011) employed by Prokka (Mirdita *et al.*, 2019). As the size of the input increases, the extra-time cost incurred by Pharokka is due to the gene prediction and tRNA calling, rather than the database searching. For extremely large metavirome datasets, Pharokka in Prodigal meta mode is therefore recommended.

In addition, for the crAss-like phage genomes, the low coding density output of some contigs identified by Pharokka indicates that stop codon reassignment may be occurring in these contigs (Peters *et al.*, 2022; Yutin *et al.*, 2021).

## Data Availability

All benchmarking input FASTA and output files, including the python script calc_gff_coding_density_prokka.py script, is available at https://doi.org/10.5281/zenodo.7227091.
